# Plant and Native Microorganisms Amplify the Positive Effects of Microbial Inoculant

**DOI:** 10.3390/microorganisms11030570

**Published:** 2023-02-24

**Authors:** Chong Li, Zhaohui Jia, Shilin Ma, Xin Liu, Jinchi Zhang, Christoph Müller

**Affiliations:** 1 Co-Innovation Center for Sustainable Forestry in Southern China, Jiangsu Province Key Laboratory of Soil and Water Conservation and Ecological Restoration, Nanjing Forestry University, 159 Longpan Road, Nanjing 210037, China; 2 Institute of Plant Ecology, Justus-Liebig University Giessen, Heinrich-Buff-Ring 26, 35392 Giessen, Germany; 3 School of Biology and Environmental Science and Earth Institute, University College Dublin, Belfield, D04V1W8 Dublin, Ireland; 4 Liebig Centre for Agroecology and Climate Impact Research, Justus Liebig University, Heinrich-Buff-Ring 26, 35392 Giessen, Germany

**Keywords:** microbial inoculant, native microorganism, plant, positive effect size

## Abstract

Microbial inoculants can be used to restore abandoned mines because of their positive effects on plant growth and soil nutrients. Currently, soils in greenhouse pot studies are routinely sterilized to eradicate microorganisms, allowing for better inoculant colonization. Large-scale field sterilization of abandoned mining site soils for restoration is difficult, though. In addition, microbial inoculants have an impact on plants. Plants also have an impact on local microbes. The interactions among microbial inoculants, native microorganisms, and plants, however, have not been studied. We created a pot experiment utilizing the soil and microbial inoculant from a previous experiment because it promoted plant growth in that experiment. To evaluate the effects of the plants, native microorganisms, and microbial inoculants, we assessed several indicators related to soil elemental cycling and integrated them into the soil multifunctionality index. The addition of the microbial inoculant and sterilizing treatment had a significant impact on alfalfa growth. When exposed to microbial inoculant treatments, the plant and sterilization treatments displayed radically different functional characteristics, where most of the unsterilized plant treatment indices were higher than those of the others. The addition of microbial inoculant significantly increased soil multifunctionality in plant treatments, particularly in the unsterilized plant treatment, where the increase in soil multifunctionality was 260%. The effect size result shows that the positive effect of microbial inoculant on soil multifunctionality and unsterilized plant treatment had the most significant promotion effect. Plant and native microorganisms amplify the positive effects of microbial inoculant.

## 1. Introduction

Mining has had significant negative impacts on 49.9 million km^2^ of terrestrial land on Earth [1], and in China, as of 2018, there were ~99,000 abandoned mines [2]. Natural revegetation and soil restoration are typically hampered by mining activities, which generally deplete topsoil and vegetation, impair soil biodiversity, and provide exceptionally harsh environmental conditions [3]. At mining sites with severe erosion, large amounts of rock are exposed; thus, the native soil has degraded or been completely decimated [4]. Spraying a mixture of grass seeds, soils, and nutrients over exposed rock surfaces has been demonstrated as an effective strategy for the initial restoration of localized ecosystems at abandoned mine sites [5]. However, because these soil mixtures have a finite nutrient load, plants stopped growing once the nutrient and water complements were depleted. Further, the sprayed seeding materials gradually separated from the rock mass [6]. To resolve this issue, previous studies undertook to screen a series of mineral-solubilizing microorganisms that might significantly increase the release of Ca and Mg. These microorganisms generated abundant acetic acid in their secretions, which consequently lowered the pH of the medium and reduced the diameter of the mineral particle sizes. Additionally, they significantly increased the concentrations of accessible nutrients in soils, which had a positive impact on plant growth [6,7,8,9,10,11,12,13]. The inoculation of these microorganisms can significantly improve the efficacy of mine site restoration.

Currently, researchers typically sterilize soil in greenhouse pot experiments to remove microorganisms, which allow for the improved colonization of inoculants [14,15,16]. However, the guest soils of abandoned mine sites for restoration are difficult to sterilize in the field. In addition, a lack of native beneficial soil microbes may significantly hinder plant growth and survival [17]. Native beneficial microbes also had a significant impact on plant nutrient uptake, the cycling of nutrients in the soil, and the production of compounds that stimulate plant growth [18]. Therefore, microbial inoculants that still functioned in the absence of sterilization in the field were critical to their usefulness, which relied on interactions with native microorganisms. Thus, we hypothesized that microbial inoculants, which functioned in the presence of native microorganisms, would continue to do so even after soil sterilization. In a previous study, microbial inoculant was observed to stimulate the soil resident microbial population for the joint promotion of plant growth [19]. Therefore, we proposed a second hypothesis that native microorganisms facilitated the more effective functioning of microbial inoculants through collaboration. Microbial inoculants have been observed to improve plant growth [7,8,9], as plants and soil microbiomes have good communication mechanisms [20]. Further, plant metabolites can selectively recruit microorganisms that can enhance plant resilience [21]. Therefore, we proposed a third hypothesis that plants, native microorganisms, and microbial inoculants create synergy, with plants and native microorganisms amplifying the positive effects of microbial inoculants. To verify our assumptions, we designed a pot experiment, using soil from an earlier experiment, and selected *Bacillus thuringiensis* NL-11 as the microbial inoculant, as it improved plant growth in the prior experiment [7]. Three factors employed for the pot experiment included plant, sterilization, and microbial inoculant treatments. We quantified a series of indices related to soil nutrients and integrated them into the soil multifunctionality index to characterize the effects of the plants, native microorganisms, and microbial inoculants [22,23]. Hedges’d was used to measure and compare the effect sizes of the microbial inoculants on the multifunctionality under the background of plants and native microorganisms. If we can prove these hypotheses, these results will be essential for the expansion of the overall mine rehabilitation industry.

## 2. Materials and Methods

### 2.1. Preparing of Experimental Materials

The entire experiment was conducted in a growth chamber (28 °C average temperature, 80% relative humidity, 16 h light/8 h dark). The *Medicago sativa* L. was picked because it is frequently utilized in the ecological restoration of mines to prepare aseptic seedlings. The seeds of *Medicago sativa* L. were purchased from the Tianhe nursery garden company (Yancheng, Jiangsu, China). The sterilized seeds were placed in a container, filled with 60 °C sterile water, and agitated for 5 min before germination. The seeds were cleaned with sterile water after soaking them in cold water for 24 h. The seeds were then combined in a 1:3 ratio with wet, sterilized sand and kept at 25 °C in a plant incubator. The seeds were covered with a wet grass tablet and left to germinate for 3–4 days. Each day, the seedlings were sprayed with warm, sterilized water (30 °C). Germinated seedlings (about 1 cm in height) were selected and transplanted into plastic pots (Ø13.8 cm × 12.5 cm deep). 

Each pot was filled with a soil mixture that included peat soil, dolostone rock powder, soil wood fiber, and organic fertilizer as nursery substrates (soil/wood fiber/organic fertilizer/peat soil/rock powder, 92:0.7:5:2:0.3). The soil was collected from the Xiashu Forestry Station, which is located at 32°7′47″ N and 119°13′15″ E. The restoration of the nearby carbonate mining areas utilized this soil as a guest soil. The soil was classified as Ultisol according to USDA Soil Taxonomy, and the soil texture was loam clay [24]. The soil had a pH of 7.25 (soil: water ratio, 1:5) and had available K and P concentrations of 100.25 mg/kg and 9.89 mg/kg, respectively. Before the pot trials, the soil was filtered using a 5 mm sieve. 

The microbial inoculant required for the experiment was *Bacillus thuringiensis* NL-11, which was isolated from the soil surrounding weathered dolostones [12]. The release of Ca and Mg significantly increased after this strain was inoculated with a mineral sample. The strain NL-11 produced a lot of acetic acid in its secretions, which evidently lowered the pH of the medium and consequently shrunk the diameter of the particle sizes. Additionally, the strain NL-11 significantly increased the amount of available phosphorus (P) and potassium (K) in soils, which had a positive impact on plant growth [8,9,10,12]. The *Bacillus thuringiensis* NL-11 was cultured at 28 °C for 24 h on Luria–Bertani agar medium (Qingdao Hope Bio-Technology Co., Ltd., Qingdao, China). A single colony from a freshly streaked plate was then selected, inoculated into Luria–Bertani broth, and incubated at 28 °C for 48 h in a shaker at 200 rpm. After 2 days, the broth with strain NL-11 was subsequently moved into the 5 L bioreactor (Sartorius BIOSTAT^®^ B Plus, Göttingen, Germany) at 28 °C. At set intervals during the fermentation process, the bacteria were extracted to determine their OD600 value. Once the change curves crested and began to decline, the bacteria were transferred to a sterile plastic bottle and maintained there [7]. Finally, the population density of *Bacillus thuringiensis* NL-11 was confirmed to be at least 1.0 × 10^8^ CFU ml^−1^ medium.

### 2.2. Experimental Design

Three factors were used in the pot experiment: the plant treatment, the sterilization treatment, and the microbial inoculant treatment. A randomized full-block design was used in the experiment, and three replicates of each treatment were used, for a total of 24 pots (Figure S1). The required soil substrates were sterilized using a high-temperature autoclave (120 °C for 70 min over two days). For unsterilization treatment, native microbial communities were collected from soils before sterilization and added to the sterilized soil in the form of a soil suspension before using microbial inoculant and seedlings. Each pot contained 800 g of soil substrate and ten seedlings. After a week, we thinned out seedlings and maintained two seedlings in each pot. For microbial inoculant treatment, 10 mL unsterilized and sterilized mediums (1.0 × 10^8^ CFU mL^−1^) were added to the pots. The experiment lasted from 15 October 2021 to 20 January 2022. Every pot was watered daily with 500 mL sterile distilled water, and destructive sampling was performed after about 90 days of plant growth. The plants were harvested and dried to constant weight. The soil samples (soil near the roots under plant treatment) were collected and stored at 4 °C for further experiments.

### 2.3. Soil Nutrient and Enzyme Activity Assay

We measured 10 soil nutrient indices related to C, N, P, S, and K. These indices were related to plant growth. The soil pH was determined at a 1:2.5 soil:solution ratio (in deionized water) using a PB-10 pH meter (Sartorius GmbH, Göttingen, Germany) after shaking for 1 h. The soil’s total N (TN), total carbon (TC), and total sulfur (TS) were estimated by an Element Analyzer (Vario MAX cube; Elementar, Germany). According to the national standard, NH_4_^+^-N and NO_3_^−^-N were extracted with 2 M KCl at a soil/extractant ratio of 1:5 after shaking for 60 min at 250 rpm and 25 °C and analyzed on a spectrophotometer (UV2700, Shimadzu, Japan) (LY/T1230-1999 and LY/T1231-1999). The available phosphorus (AP) was extracted with 0.5M sodium bicarbonate and determined by using the molybdate-blue colorimetric method (LY/T1230-1999). The available potassium (AK) was extracted with 1 M ammonium acetate and determined by flame photometer (LY/T1236-1999). The total potassium (TK) was also determined by a flame photometer (LY/T1234-1999). Soil organic carbon (SOC) was quantified with the potassium dichromate oxidation external heating method (HJ615-2011).

We also detected 10 extracellular soil enzymes involved in elemental cycling. These indicators were C-cycling enzymes (β-1,4-glucosidase, invertase, polyphenol oxidase, and peroxidase), N-cycling enzymes (β-1,4-N-acetylglucosaminidase and urease), P-cycling enzymes (acid, neutral, and alkaline phosphatase), and S-cycling enzyme (arylsulfatase). The β-1,4-glucosidase activity was assayed using p-Nitrophenyl-β-D-glucopyranoside as a substrate. Soil invertase activity was tested using the 3,5-dinitro salicylic acid (DNS) method. The peroxidase and polyphenol oxidase activities were detected by iodometry. The β-1,4-N-acetylglucosaminidase activity was assayed by an improved colorimetric determination of the intensity of the yellow color produced by p-nitrophenyl release. The urease activity was quantified using steam distillation methods with urea as substrate. The phosphatase activity was tested using a disodium phenyl phosphate solution as a substrate. Arylsulfatase was assayed by measuring the released nitrophenol from nitrophenol potassium sulfate.

### 2.4. Statistical Analysis

Soil nutrients and enzyme activities were Z-score-transformed, and a heat map was generated by GraphPad Prism 9 (GraphPad Software, Inc., La Jolla, CA, USA). The coefficients of variation (CV) of the individual soil enzyme activities were then calculated. We assessed soil multifunctionality using the averaging method. To create a multifunctionality index, the standardized Z-score rates of the soil nutrients and enzyme functions were averaged [23]. 

To compare the effect size of microbial inoculant on multifunctionality, we measured effect size using Hedges’d. Hedges’d is a unit-free index that estimates the size of the impact. It ranges from −∞ to +∞, where 0 denotes that there is no difference in the measured variable between the inoculation treatment and the noninoculation treatment; a negative value indicates that the inoculation treatment has a lower value than the noninoculation treatment, and a positive value presents that the inoculation treatment has a greater value than the noninoculation treatment [25]. Hedges’d was calculated as [26].

(1) $$d=\frac{{\bar{X}}^{i}-{\bar{X}}^{n}}{S}J$$

where *J* is a weighting factor based on the number of replicates (N) per pair of soil multifunctionality index variables, calculated as

(2) $$J=1-\frac{3}{4\left({\tilde{n}}^{i}+{\tilde{n}}^{n}-2\right)-1}$$

and *S* is the pooled standard deviation based on the standard deviations (*s*) per pair of soil multifunctionality index variables, calculated as

(3) $$S=\sqrt{\frac{\left({\tilde{n}}^{i}-1\right){\left(s^{i}\right)}^{2}+\left({\tilde{n}}^{n}-1\right){\left(s^{n}\right)}^{2}}{{\tilde{n}}^{i}+{\tilde{n}}^{n}-2}}$$

The variance of Hedges’d was calculated as:

(4) $$v_{d}=\frac{{\tilde{n}}^{i}+{\tilde{n}}^{n}}{{\tilde{n}}^{i}{\tilde{n}}^{n}}+\frac{d^{2}}{2\left({\tilde{n}}^{i}+{\tilde{n}}^{n}\right)}$$

where ${\bar{X}}^{i}$ and ${\bar{X}}^{n}$ are the mean values of the microbial inoculant and control group and $s^{i}$, $s^{n}$, ${\tilde{n}}^{i}$, and ${\tilde{n}}^{n}$ are the standard deviations and sample sizes for the microbial inoculant and control group.

All calculations on effect size were performed in Metawin (Version 2.1; Sinauer Associates, Inc., Sunderland, MA, USA) software.

Data analysis was performed using Microsoft Excel and the SPSS package (version 21.0, IBM, Armonk, NY, USA), and the data were expressed as a mean ± standard deviation (SD). We analyzed data for normality and homogeneity and confirmed that the assumptions of normality and homogeneity were met, and the graphs were developed by GraphPad Prism 9 (GraphPad Software, Inc., La Jolla, CA, USA). A nonmetric multidimensional scaling (NMDS) analysis based on the Bray–Curtis dissimilarity was performed to explore any differences in the plant, sterilization, and microbial inoculant treatments. Pearson’s correlation analysis was performed to explain the correlation between soil nutrients and enzyme activities. The significance of different treatments on various indices was evaluated by ANOVA analysis with LSD’s multiple comparisons, taking *p* ≤ 0.05 as a significance level.

## 3. Results

**Figure 1:**
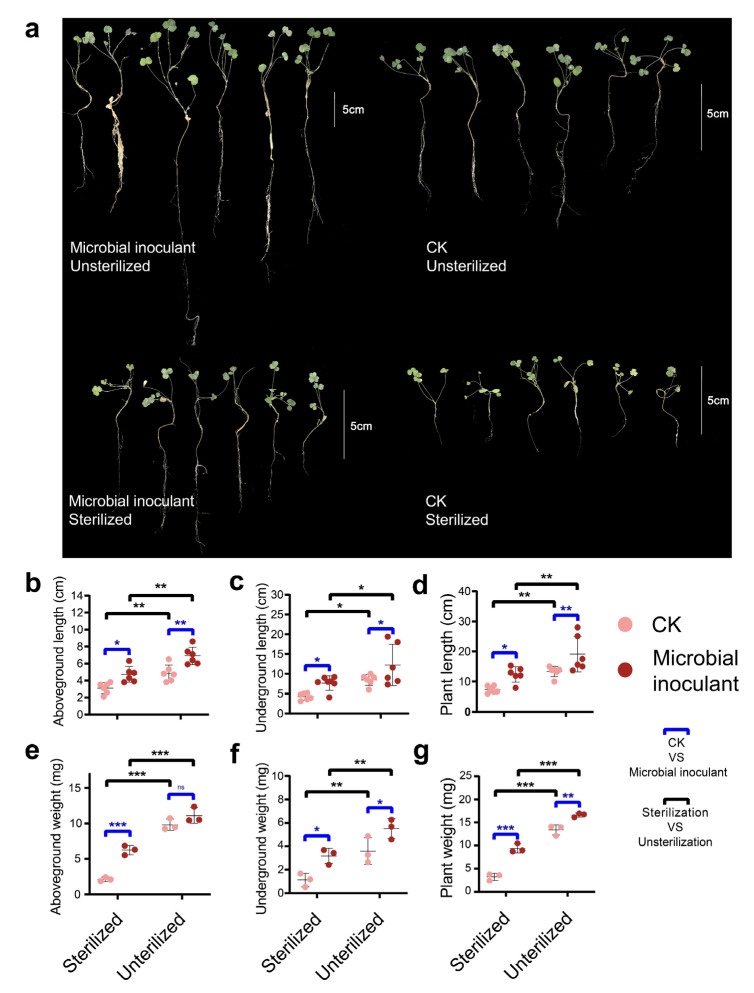
(**a**) Plant growth under different treatments. (**b**) aboveground length. (**c**) underground length. (**d**) plant length. (**e**) aboveground dry weight. (**f**) underground dry weight. (**g**) plant weight. Note: ns indicates no significant effect, * indicates *p <* 0.05, ** indicates *p <* 0.01, and *** indicates *p <* 0.001.

**Figure 2:**
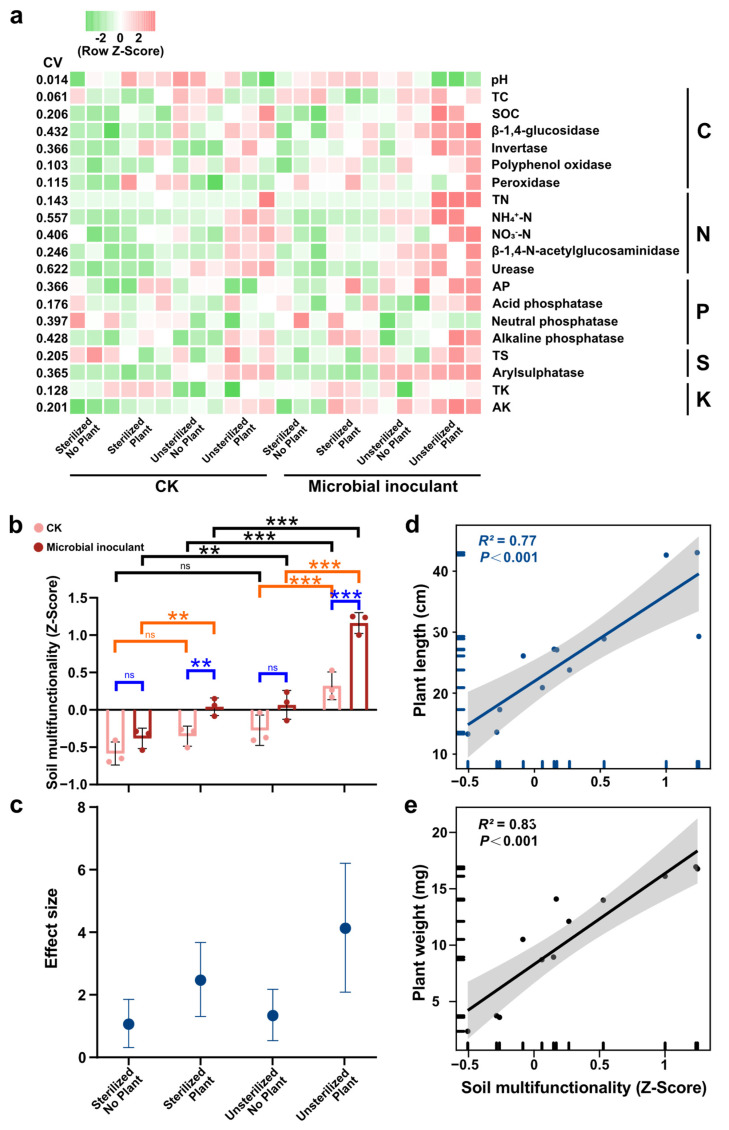
(**a**) Heat map showing the change in soil nutrients and enzyme activities under different treatments. (**b**) soil multifunctionality indices under different treatments. (**c**) the effect size of microbial inoculant on soil multifunctionality under the background of the plant and native microorganisms. (**d**,**e**) correlation of soil multifunctionality with plant length and dry weight. Note: ns indicates no significant effect, ** indicates *p <* 0.01, and *** indicates *p <* 0.001.

The addition of microbial inoculant and sterilizing treatment had a significant impact on alfalfa growth (*p* < 0.05) (Table S1). With the addition of the microbial inoculant, plant length and dry weight were significantly increased (*p* < 0.05). The sterilizing treatment resulted in a significant decrease in plant length and dry weight (*p* < 0.05) (Figure 1). When exposed to microbial inoculant treatments, the plant and sterilization treatments displayed radically different functional characteristics, whereas most of the unsterilized plant treatment indices were higher than those of the others (Figure 2a, Figures S2 and S3). In particular, under the background of the presence of plants and native microorganisms, the microbial inoculant significantly increased soil nitrate nitrogen by 28.5%, total carbon by 12.2%, total nitrogen by 20%, available potassium by 17.2%, available phosphorus by 100%, ammonium nitrogen by 39%, organic carbon by 20.3%, β-1,4-glucosidase by 27.5%, β-1,4-N-acetylglucosaminidase by 27.1%, urease by 25.1%, peroxidase by 20.3%, arylsulfatase by 10.4%, and alkaline phosphatase by 37.9%. Microbial inoculants, however, did not significantly increase the activities of soil nutrients and enzymes in other situations (Figure 2a, Figures S2 and S3). Plant treatment significantly improved soil NO_3_^−^-N, total carbon, accessible potassium, NH_4+_-N, -1,4-N-acetylglucosaminidase, invertase, urease, acid phosphatase, alkaline phosphatase, and arylsulfatase when there were native microorganisms present. The effects of plant treatment on the majority of soil nutrients and enzyme activity were not significant in the absence of natural microorganisms (Figures S2 and S3). Nonmetric multidimensional scaling (NMDS) based on the indices of all soil nutrient and enzyme activities was performed to elucidate the changes in soil functional traits among the various treatments (Figure S4). Three nonparametric multivariate analyses of dissimilarity, including the nonparametric multivariate analysis of variance (Adonis), analysis of similarity (ANOSIM), and multiresponse permutation procedure (MRPP) demonstrated significant differences among the different treatments (Figure S4a). Notable differences in multifunctional soil nutrient and enzyme activity indices were also found among the different treatments (Table S1 and Figure S4b).

To better compare the effects of microbial inoculation, and plant and sterilization treatments on soil nutrients and enzyme activities, we integrated them into the soil multifunctionality index. The results show that the addition of microbial inoculant significantly increased soil multifunctionality in plant treatments, particularly in the unsterilized-plant treatment, where the increase in soil multifunctionality was 260% (*p* < 0.05) (Figure 2b). In the absence of plants, the promotion effect of microbial inoculants on soil multifunctionality was not significant (*p* > 0.05). Plants significantly enhanced soil multifunctionality in the context of microbial inoculation or in the presence of native microorganisms (*p* < 0.05) (Figure 2b). Sterilization treatment also significantly decreased soil multifunctionality, particularly in the absence of plants. Additionally, the soil multifunctionality index was positively correlated with most soil nutrient and enzyme activity indices (Figure S5), including soil organic carbon, β-1,4-glucosidase, total N, NH_4_^+^-N, NO_3_^−^-N, β-1,4-N-acetylglucosaminidase, urease, alkaline phosphatase, arylsulfatase, and available potassium, and significantly positively correlated with plant length and dry weight (*p* < 0.001) (Figure 2d,e). The effect size result shows that the positive effect of the microbial inoculant on soil multifunctionality, and unsterilized plant treatment had the most significant promotion effect (Figure 2c).

## 4. Discussion

The plant growth results demonstrate that microbial inoculants and sterilization affected plant growth (Table S1). Microbial inoculants promoted plant growth in both nonsterile and sterilized soils (Figure 1), which supported our first hypothesis. In terms of plant–microbe interactions, Bacillaceae microorganisms have been found in numerous plant rhizospheres, which are well known for their abilities to protect plants from illness and stimulate plant development [27,28]. For some experiments, in the absence of native soil microorganisms, Bacillaceae were shown to directly enhance plant growth through phytohormone production [29,30] and phosphate solubilization [12,31], etc. Another finding demonstrated that unsterilized soil promoted more efficient plant growth in contrast to sterilized soil (Figure 1). This was because plants recruited beneficial microbes from the soil that were linked to biogeochemical cycles, which could enhance soil fertility and improve the nutritional status of plants [32]. Plants benefit from these microbes either directly or indirectly through a range of mechanisms, including as nutritional assistance (facilitating nutrient uptake from the soil) and growth stimulation via phytoregulator production [33,34]. However, a previous study had shown that various bacteria, fungi, and even mycorrhizal fungi can have negative effects on plant growth [17,35]. This might be that the influence on plants is ultimately determined by the positive and negative interactions between root-associated microorganisms. In the present study, the positive effects of native microorganisms on plants dominated, which in turn led to positive feedback effects on the microbial community [36]. These results also reveal that maximum plant growth was made possible through the combination of native microorganisms and microbial inoculants, which supported our second hypothesis. This phenomenon was due to the capacity of microbial inoculants to function more effectively through synergistic cooperation with specific native microorganisms. In a previous study, microbial inoculant stimulated a soil resident *Aspergillus* population for the joint promotion of plant growth [19]. *Bacillus* also had synergistic effects with rhizobia, which boosted the symbiotic nodulation of rhizobia and legumes to influence plant growth [37].

Plant growth was intimately related to changes in soil nutrients [38], and soil enzymes also played important roles in the cycling of nutrients [39]. Consequently, for this study, we identified 20 soil nutrients and enzymes involved in elemental cycling. The results indicate that different treatments translated to distinct functional traits (Figure 2a), where most of the unsterilized plant treatment indices were higher than those of the others (Figures S2 and S3). Nonmetric multidimensional scaling (NMDS) based on the indices of all soil nutrient and enzyme activities was performed to elucidate the changes in soil functional traits among the various treatments (Figure S4). Notable differences in multifunctional soil nutrient and enzyme activity indices were found among the different treatments (Figure S4). These results demonstrate that plants, sterilization, and microbial inoculants affected both soil nutrients and enzyme activities (Table S1). Additionally, most soil nutrients and enzyme activities were positively correlated (Figure S5), which indicated that plants, native microorganisms, and microbial inoculant-mediated nutrients and enzyme activities were closely related.

A soil multifunctionality index was employed to integrate the activities of soil nutrients and enzymes, which were positively correlated with most soil nutrient and enzyme activity indices (Figure S5), and significantly positively correlated with plant growth (*p* < 0.001) (Figure 2d,e). This demonstrated that changes in microbial inoculant and native microorganism-mediated soil multifunctionality were the main sources of changes in plant growth, which was consistent with earlier conclusions [7,10]. 

The effects of microbial inoculants, sterilization, and plants on soil multifunctionality are shown in Figure 2b. The results reveal that microbial inoculants had no significant effects on the multifunctionality of soil in the absence of plants (*p* > 0.05), while in their presence, there was a significant impact (*p* < 0.01) (Figure 2b), which might have had to do with plant–soil feedback [38]. The microbial inoculant improved the growth of plants, which modified their soil environment including the availability of nutrients [40] that fed back on the growth of seedlings and their survival [41]. Plant can also allocate carbohydrates to microorganisms [42]. In the absence of plants, most functions of the microbial inoculant that promoted plant growth were disabled and much less effective, which terminated the benign plant–soil feedback. Additionally, in the absence of microbial inoculant and in the presence of native microorganisms, there was no significant difference between plant and nonplant treatments (Figure 2b). It demonstrated the significance of soil microorganisms for plant development and how interactions between plants and microbes in the rhizosphere affect plant health and soil fertility [43]. Further, the enhancement of soil multifunctionality by native microorganisms indicated that they induced soil multifunctionality [22,44,45]. Our results reveal that soil multifunctionality under the microbial inoculation and unsterilized plant treatment was highest, which supported our third hypothesis. This highlighted how native microorganisms and microbial inoculants can cooperate to influence plants in beneficial ways. Once plant growth has been stimulated, they release signals that they then use to feed back into the soil. Thus, a positive feedback loop is created, and the effect of one plus one is greater than two. The result of the effect size was also consistent with this (Figure 2c), where plants and native microorganisms amplified the positive effects of the microbial inoculant.

Through this experiment, we demonstrated that native microorganisms and plants improved the efficacy of microbial inoculants. Microbial inoculants would, however, compete with native species for resources as invaders [46]. Competition might impact an invader’s ability to survive, which would ultimately result in different invasion impacts [47]. The response of the native soil microbial community to the introduction of a microbial inoculant is unclear. *Bacillus thuringiensis* NL-11 was chosen as the microbial inoculant in this study because it enhanced plant growth in the previous experiment. However, strain NL-11 may or may not still be effective in various soil types or environments. For future ecological mine site restoration projects, we aim to screen groups of functional microorganisms for specific mine types and site conditions worldwide to develop a global microbial inoculant library. For abandoned mines at the regional level, different microbial inoculants from the microbial inoculant library and plant seeds will be inoculated into local mine soils in the greenhouse to identify their optimal combination for application in the field for mine restoration. This will likely enhance the effectiveness of whole mine restoration, which is of great importance for the development of the overall mine rehabilitation industry.

## 5. Conclusions

In this study, microbial inoculants that worked while native microorganisms were present would still work even after soil sterilization. The native microorganisms facilitated the more effective functioning of microbial inoculants through collaboration. Plants, native microorganisms, and microbial inoculants created synergy, with plants and native microorganisms amplifying the positive effects of microbial inoculants. In general, native microorganisms and plants are essential to the function of microbial inoculants, and we should take these elements into account in our future uses of microbial inoculants.

## Data Availability

The data that support the findings of this study are available in Figshare with the identifier https://figshare.com/account/home#/projects/147141, accessed on 21 February 2023.

## Supplementary Materials

The following supporting information can be downloaded at: https://www.mdpi.com/article/10.3390/microorganisms11030570/s1.
