# Supplementary material for: Plant and Native Microorganisms Amplify the Positive Effects of Microbial Inoculant
Source: Microorganisms. 2023 Feb 24;11(3):570. doi: 10.3390/microorganisms11030570 (PMC10055949; doi:10.3390/microorganisms11030570)

Table. S1. One-way ANOVA analysis (*p* values) of three factors (sterilization, plant, and microbial inoculant) on soil nutrient and enzyme activities. Note: ns indicates no significant effect, * indicates $p < 0.05$, **indicates $p < 0.01$, and ** indicates $p < 0.001$.

	Sterilization	Plant	Microbial inoculant	Sterilization× Plant	Sterilization× Microbial inoculant	Plant× Microbial inoculant	Sterilization× Plant× Microbial inoculant
Aboveground length	***	×	***	×	ns	×	×
Underground length	**	×	**	×	ns	×	×
Plant length	***	×	**	×	ns	×	×
Aboveground dry weight	***	×	***	×	*	×	×
Underground dry weight	**	×	**	×	ns	×	×
Plant dry weight	***	×	***	×	*	×	×
pH	ns	ns	ns	***	ns	ns	ns
Total carbon	**	**	**	*	ns	**	****
Soil organic carbon	***	***	ns	ns	ns	*	ns
β-1,4-glucosidase	***	**	*	ns	ns	ns	ns
Invertase	***	ns	*	ns	ns	ns	ns
Polyphenol oxidase	*	ns	*	ns	v	ns	ns
Peroxidase	ns	ns	*	ns	ns	ns	ns
Total nitrogen	**	ns	ns	**	ns	ns	ns
NH_4^+-N	***	***	***	***	***	ns	ns
NO_3^--N	**	***	ns	*	ns	ns	ns
β-1,4-N-acetylglucosaminidase	***	***	***	*	*	ns	ns
Urease	***	**	ns	*	ns	ns	ns
Available phosphorus	ns	ns	*	ns	ns	ns	ns
Acid phosphatase	ns	**	ns	*	ns	ns	ns
Neutral phosphatase	**	ns	ns	ns	ns	ns	ns

Alkaline phosphatase	ns	***	**	***	ns	ns	ns
Total sulfur	ns	ns	ns	**	ns	ns	ns
Arylsulphatase	***	**	***	***	***	ns	ns
Total potassium	ns	ns	ns	ns	ns	ns	ns
Available potassium	***	***	***	ns	ns	ns	ns
Soil multifunctionality	***	***	***	**	*	*	ns

Fig.S1. Experimental design

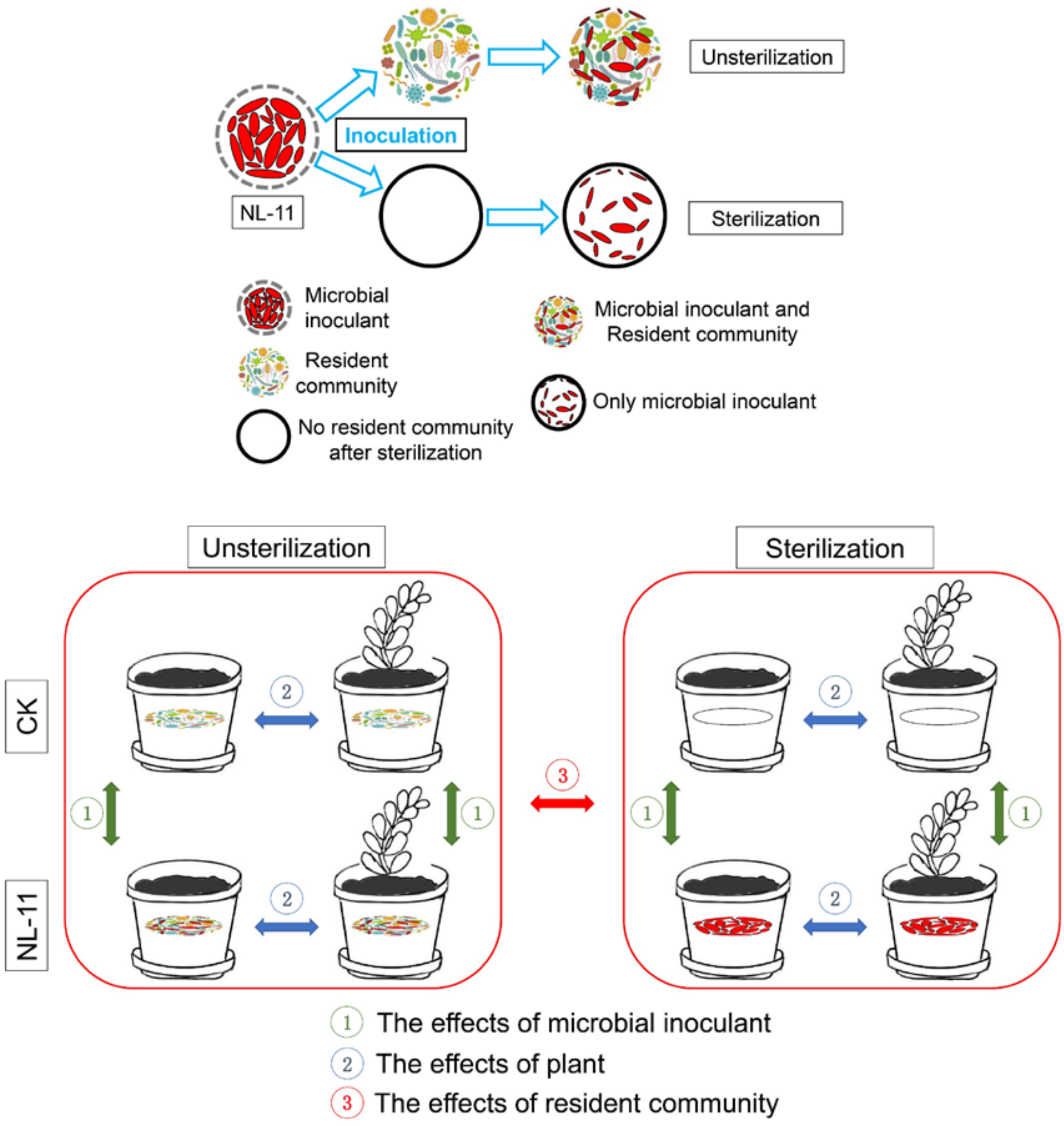

Fig. S2. Soil nutrient indexes in response to plant, sterilization, and microbial inoculant treatments. Note: ns indicates no significant effect, * indicates $p < 0.05$, **indicates $p < 0.01$, and ** indicates $p < 0.001$.

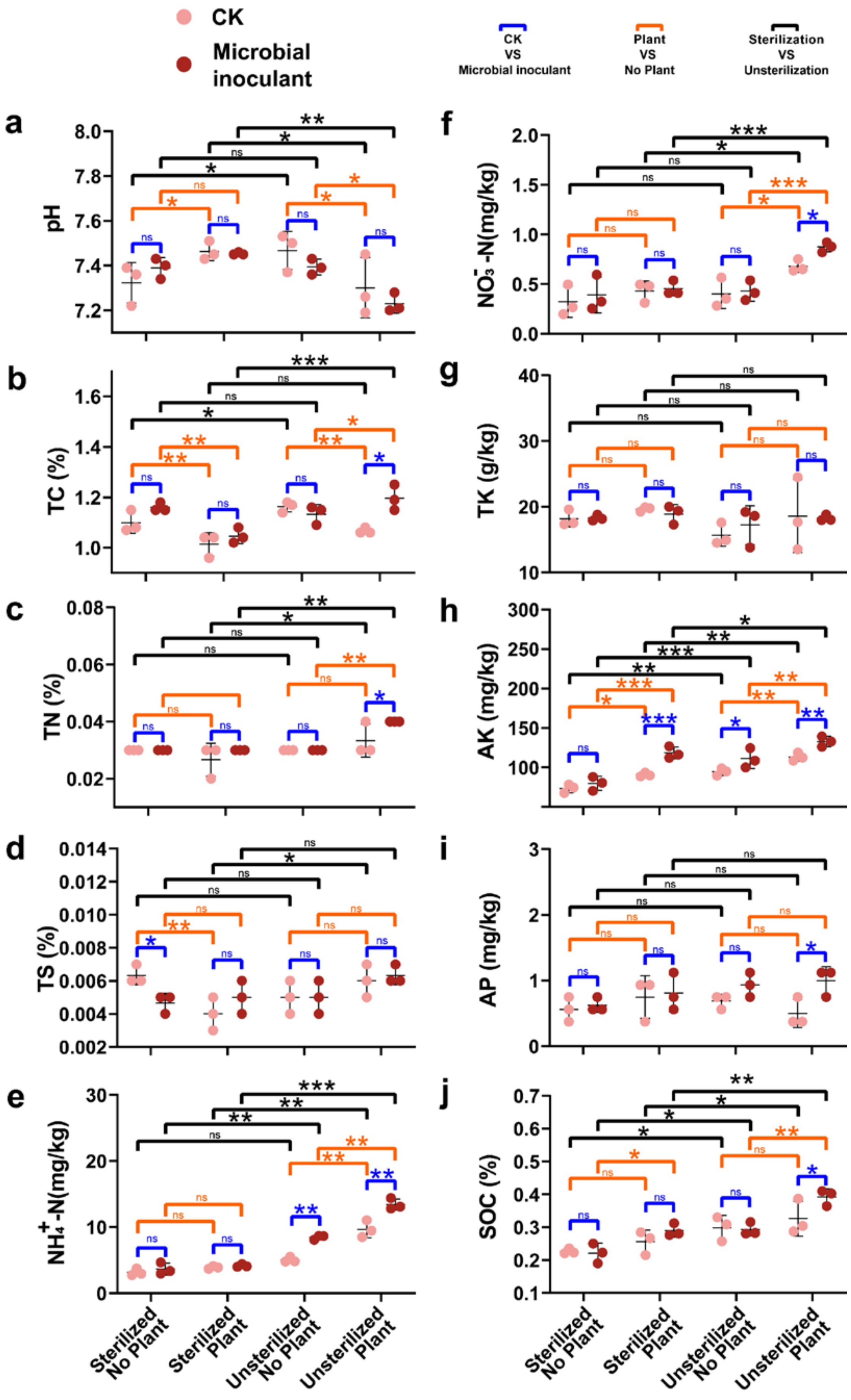

Fig. S3. Soil enzyme activity indexes in response to plant, sterilization, and microbial inoculant treatments. Note: ns indicates no significant effect, * indicates $p < 0.05$, **indicates $p < 0.01$, and *** indicates $p < 0.001$.

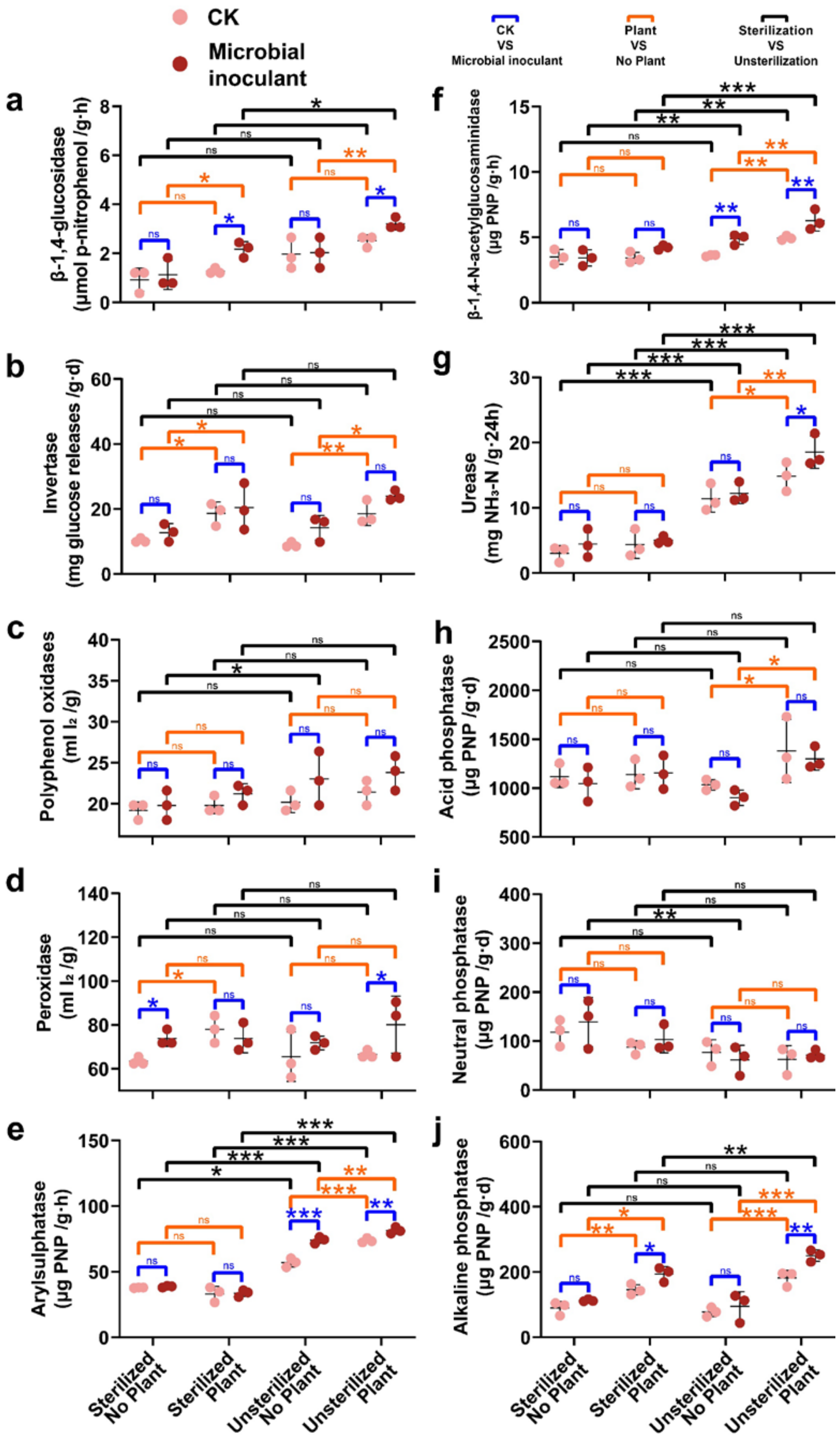

Fig. S4. Nonmetric multidimensional scaling (NMDS) analysis was based on Bray-Curtis dissimilarity.

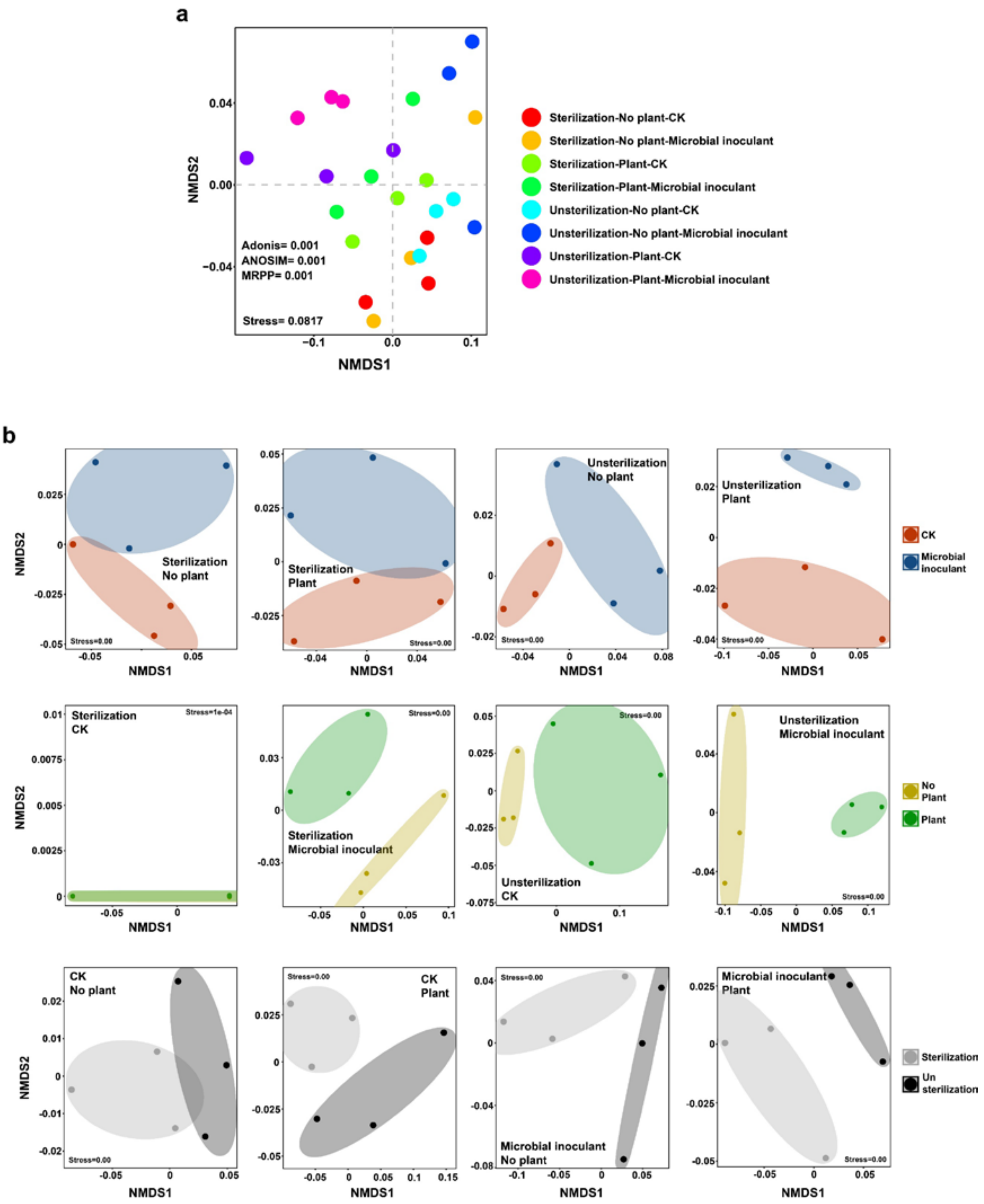

Fig. S5. The relationship among soil multifunctionality index, soil nutrient, and enzyme activity. In the square, the blue and red colors represent a positive and negative correlation, respectively, between the two variables. The association between the variables is stronger the deeper the hue.

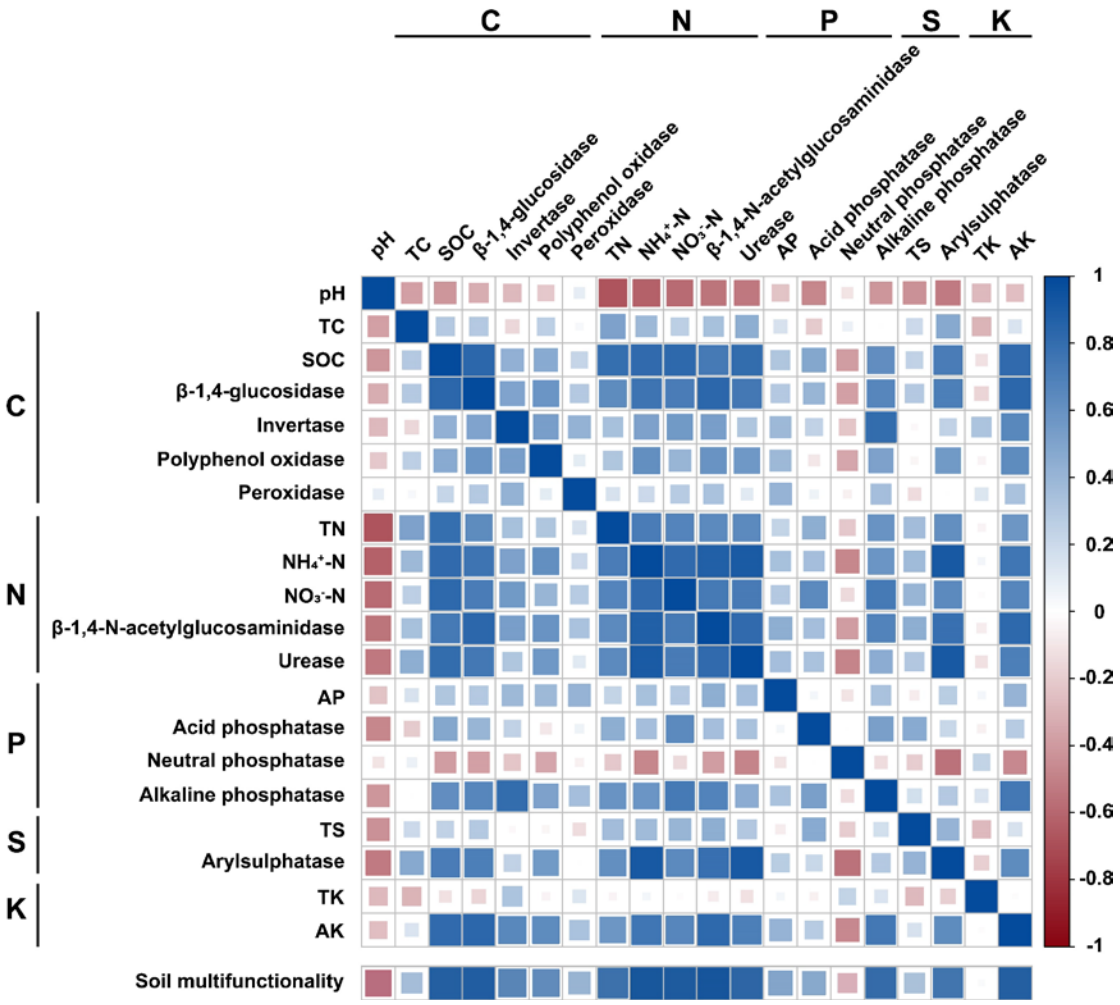

Supplement: Supplementary file 1 [file microorganisms-11-00570-s001.zip › microorganisms-2202044-supplementary.pdf]
